# Judgement bias in pigs is independent of performance in a spatial holeboard task and conditional discrimination learning

**DOI:** 10.1007/s10071-017-1095-5

**Published:** 2017-05-15

**Authors:** Sanne Roelofs, Eimear Murphy, Haifang Ni, Elise Gieling, Rebecca E. Nordquist, F. Josef van der Staay

**Affiliations:** 1 Behaviour and Welfare Group (Formerly: Emotion and Cognition Group), Department of Farm Animal Health, Faculty of Veterinary Medicine, Utrecht University, Yalelaan 7, 3584 CL Utrecht, The Netherlands; 2 Brain Center Rudolf Magnus, Utrecht University, Stratenum Building, Room STR5.203, Universiteitsweg 100, 3584 CG Utrecht, The Netherlands; 3 Division of Animal Welfare, VPHI Vetsuisse Faculty, University of Bern, Länggassstrasse 120, 3012 Bern, Switzerland; 4 Department of Methodology and Statistics, Faculty of Social and Behavioural Sciences, Utrecht University, Padualaan 14, 3584 CH Utrecht, The Netherlands

**Keywords:** Cognitive holeboard, Working memory, reference memory, Conditional discrimination, Cognitive judgement bias, Learning, Pig

## Abstract

Biases in judgement of ambiguous stimuli, as measured in a judgement bias task, have been proposed as a measure of the valence of affective states in animals. We recently suggested a list of criteria for behavioural tests of emotion, one of them stating that responses on the task used to assess emotionality should not be confounded by, among others, differences in learning capacity, i.e. must not simply reflect the cognitive capacity of an animal. We performed three independent studies in which pigs acquired a spatial holeboard task, a free choice maze which simultaneously assesses working memory and reference memory. Next, pigs learned a conditional discrimination between auditory stimuli predicting a large or small reward, a prerequisite for assessment of judgement bias. Once pigs had acquired the conditional discrimination task, optimistic responses to previously unheard ambiguous stimuli were measured in the judgement bias task as choices indicating expectation of the large reward. We found that optimism in the judgement bias task was independent of all three measures of learning and memory indicating that the performance is not dependent on the pig’s cognitive abilities. These results support the use of biases in judgement as proxy indicators of emotional valence in animals.

## Introduction

Cognitive processes and emotional processes are closely related. Although originally considered separate disciplines, the interaction between emotion and cognition has been demonstrated in both human and animal research (Lazarus 1982; Dolcos 2015). Cognitive processes are those that involve some form of information processing in the brain, such as memory, attention, problem-solving and planning (Pessoa 2008). Some authors divide cognition into lower- and higher-level cognitive processes with the higher-level ones including judgement, reasoning and decision-making (Blanchette and Richards 2010). Emotions are adaptive processes linked to the avoidance of harm and the seeking out of valuable resources, which are reflected by cognitive, behavioural and/or physiological changes (Paul et al. 2005). Since the ‘functional relationships between cognition and emotion are bidirectional’ (Lazarus 1991, p. 353), the link between emotion and cognition can be studied in different ways; how emotion affects cognitive processes or how cognition can impact upon emotional responses.

Biases in judgement of ambiguous stimuli have recently been proposed as a method for establishing the valence of emotional states in animals; optimistic judgements are said to indicate positively valenced emotions, while pessimistic judgements indicate negative emotions (Harding et al. 2004; Paul et al. 2005; Mendl et al. 2009). This approach is receiving increasing attention in animal welfare science as suggested by the number of papers and variety of species to which it has been applied (Roelofs et al. 2016). For example, treatments such as cage enrichment (Burman et al. 2008; Brydges et al. 2011), social stress (Papciak et al. 2013) and unpredictable mild stress (Harding et al. 2004; Novak et al. 2016) influence judgement bias in rodents. We recently proposed a list of criteria for behavioural tests of emotion, one of which is that a task should ‘specifically and unambiguously capture emotion-related behaviours’ (Murphy et al. 2014, p. 27). This means that responses on the task should not be confounded by differences in learning capacity, activity levels or motivation. Although the judgement bias task assesses emotion through cognitive processes, its results must not simply reflect the cognitive capacities of an animal.

Judgement bias can be measured in animals by training them to discriminate between two stimuli: one predicting a positive outcome and the other predicting a negative or less positive outcome (Mendl et al. 2009; Roelofs et al. 2016). Animals are trained to display a specific behaviour in response to each of these cues. After successful discrimination training, the animal is presented with ambiguous stimuli, often with qualities intermediate between the positive and negative cues. Then, the behavioural responses to the ambiguous stimuli are taken to indicate an animal’s expectation of the positive or negative outcome (Mendl et al. 2009).

We have developed an active choice task to assess judgement bias in pigs (Murphy et al. 2013a, b, 2015; Roelofs et al. 2017). Prior to judgement bias testing, pigs are trained in an audio-spatial conditional discrimination task. Tone-cues of different frequencies are used as positive and negative stimuli, predicting the presence of either a large food reward (positive outcome) or a small food reward (negative outcome) in a left or right goal-box. Put simply, the pigs need to learn: if the positive tone-cue, predicting a large reward, is presented, go to the left goal-box; if the negative tone-cue, predicting a small reward, is presented, go to the right goal-box (Murphy et al. 2013a). Once pigs reach a predefined criterion level of performance on the conditional discrimination task, a series of ambiguous tone-cues is presented, of intermediate frequencies between the previously trained tones. Responses indicating expectation of the large reward (i.e. approaches to the ‘positive’ goal-box) are considered optimistic responses and recorded as a measure of judgement bias.

Spatial holeboard tasks have been established as valid instruments in cognitive research as they allow measuring multiple facets of cognition simultaneously in one test (van der Staay et al. 2012). The holeboard is a free choice maze for assessing spatial learning and memory. It contains a number of ‘holes’, potential reward locations, of which only a subset is baited. An animal can search freely for the baited holes within a certain time period. The holeboard allows for the assessment of both working memory and reference memory. Working memory is a short-term memory that contains information which is only relevant within a testing trial (Dudchenko 2004). In the holeboard, working memory allows animals to remember which holes have already been visited during a trial, so unrewarded revisits of holes are avoided. This information is no longer relevant once the trial has ended (Dudchenko 2004; van der Staay et al. 2012). Reference memory is a long-term memory that contains ‘the general rules of a task’ (van der Staay et al. 2012, p. 383). This information remains relevant across trials. In the holeboard, reference memory allows animals to remember which subset of holes is baited.

The holeboard task was first adapted for use with pigs by Arts et al. (2009), who found that it was suitable for measuring spatial learning in this species. Further studies have validated the suitability of the holeboard for simultaneous measurement of task-specific reference memory and trial-specific working memory in pigs (Gieling et al. 2012, 2013, 2014; Bolhuis et al. 2013; Haagensen et al. 2013a, b; Antonides et al. 2015; Fijn et al. 2016; Roelofs et al. 2017).

Cognitive performance in pigs can be described by different measures, examples of which are working and reference memory in spatial learning tasks, or auditory-spatial memory in the conditional discrimination task preceding judgement bias testing. Therefore, when assessing whether judgement bias is independent of cognitive abilities, it is preferable to include a variety of cognitive measures in the analysis. This requires animals that have been subjected to multiple learning and memory tasks (Zanghi et al. 2015). In previous experiments, no correlation was found between pigs’ performance in conditional discrimination training prior to judgement bias testing, and optimism measured in the judgement bias task (Murphy et al. 2013b). Also, no correlation was found between performance in a pig gambling task (measuring decision-making under risk) and optimism in the judgement bias task (Murphy et al. 2015).

The question whether measures of spatial working and reference memory in holeboard-type tasks are independent has been addressed in a similar manner in only a small number of studies. No correlation was found between working and reference memory of rats trained in the holeboard or cone field, a modification of the holeboard (van der Staay et al. 1990; Blokland et al. 1992; Prickaerts et al. 1999; van der Staay 1999). Further evidence supporting this notion comes from a factor analysis of the cognitive and behavioural performance of inbred mice in the modified holeboard (Ohl et al. 2003). This study revealed that working and reference memory loaded on different factors. Separate factor analyses performed in an age-comparison study of C57/BL mice yielded evidence that working memory and reference memory were independent in the oldest (24-month-old), but not in middle-aged (16-month-old) or young adult (4-month-old) mice (Weiss et al. 1998).

A problem with these findings is that a lack of correlation between variables does not provide evidence for their independence. Standard statistical analysis provides a *p* value which, when it falls below a specified threshold (often 0.05), allows for the rejection of the null hypothesis in favour of the alternative hypothesis. Unfortunately, a *p* value of >0.05 does not provide evidence for the null hypothesis (Wagenmakers 2007; Rouder et al. 2009). In the case of correlation analysis, the null hypothesis stating that there is no correlation between variables cannot be proven using standard tests. To provide evidence for independence of variables (shown by a lack of correlation), Bayesian statistics are preferable, as these provide an estimate of support for the null hypothesis (Gallistel 2009; Wetzels and Wagenmakers 2012; Wagenmakers et al. 2016).

In order to assess whether optimism of pigs in an active choice judgement bias task was related to their learning ability, we used the results of three studies in which pigs were first tested in the spatial holeboard task (Gieling et al. 2013, 2014; Roelofs et al. 2017), followed by the judgement bias task (Murphy et al. 2013b; Roelofs et al. 2017). Firstly, we expect that optimism, as measured in the judgement bias task, will be found to be independent of learning ability, as measured by working and reference memory performance in the holeboard task and the acquisition of the conditional discrimination task preceding judgement bias testing. Second, we expect that working and reference memory as measured in the spatial holeboard task will be independent of one another, i.e. that they represent different memory domains, as shown previously in studies using rodents as subjects. Third, reference memory may be important for successful performance in conditional discrimination training as well as the spatial holeboard. During discrimination training, the animals have to memorize general rules of the task, for example: ‘if cue A, response A; if cue B, response B’ (Murphy et al. 2013a). Therefore, we expect that reference memory in the spatial holeboard task and acquisition of the conditional discrimination task are correlated.

## Materials and methods

The animals and methods used in this study are described in detail elsewhere: Experiment 1 in Gieling et al. (2013) and Murphy et al. (2013a, b); Experiment 2 in Gieling et al. (2014) and Murphy et al. (2015); Experiment 3 in Roelofs et al. (2017).

### Subjects and Housing

Information on the subjects used in each study is provided in Table 1. Shortly after weaning at approximately 4 weeks of age, pigs were moved to the research stables. Pigs were group housed in two straw-bedded pens (15–20 m^2^, 8–10 pigs per pen) in naturally lit and ventilated stables. Each pen contained a covered nest area and was provided with toys for enrichment. Pigs had access to water ad libitum and were fed twice per day (Holeboard: 33% morning, 66% evening; Judgement bias task: 25% morning, 75% evening), amounts according to the recommendations of their breeders.

**Table 1:** Overview of subjects used, age in weeks at start of holeboard task training and conditional discrimination training preceding the judgement bias task and number of trials (trial blocks) of the acquisition of the holeboard task in studies 1, 2 and 3. Habituation sessions and pre-training sessions preceded formal training (not included in table)

Study	Subjects	*N*; Sex	Breed	Supplier	Approximate age in weeks at start of formal training/testing			Trials (trial blocks) in acquisition phase
					HBT	CDT	JBT^§^	HBT
1	Miniature pigs	8 ♀	Göttingen miniature pigs	Ellegaard, Denmark	13	26	28	104 (26)
	Conventional pigs	7 ♀	Duroc × Yorkshire and Duroc × Danish Landrace mix	Utrecht University	13	26	28	104 (26)
2^a^	LBW* Allopurinol^†^	10; 4♀, 6♂	(Terra × Finnish landrace) × Duroc mix	Utrecht University	8	20	24.5	40 (10)
	LBW* Control^†^	8; 1♀, 7♂	(Terra × Finnish landrace) × Duroc mix	Utrecht University	8	20	24.5	40 (10)
	NBW* Allopurinol^†^	10; 5♀, 5♂	(Terra × Finnish landrace) × Duroc mix	Utrecht University	8	20	24.5	40 (10)
	NBW* Control^†^	9; 1♀, 8♂	(Terra × Finnish landrace) × Duroc mix	Utrecht University	8	20	24.5	40 (10)
3	Female pigs^‡^	10 ♀	(Yorkshire × Finnish Landrace) × Duroc mix	Utrecht University	9	21	28	40–60 (10–15)
	Male pigs^‡^	10 ♂	(Yorkshire × Finnish Landrace) × Duroc mix	Utrecht University	9	21	28	40–60 (10–15)

*HBT* holeboard task, *CDT* conditional discrimination task, *JBT* judgement bias task

* All piglets from 10 litters were weighed at birth and the litter mean calculated. Low-birth-weight (LBW) pigs weighed at least 1SD below the litter mean, while normal-birth-weight (NBW) pigs were those closest to an adjusted litter mean (mean excluding LBW pig weights) as in Gieling et al. (2014)

^†^ Five sows had been treated with 15 mg kg^−1^ Allopurinol for the 30 days (±2 days) before farrowing

^‡^ All piglets from 8 litters were weighed at birth. One or two male piglets and one or two corresponding female piglets weighing closest to the litter mean (calculated separately for male and female piglets) were selected from each litter

^§^ As pigs learned at different rates, this age refers to the mean age at starting the JBT

^a^Performed in two replicates

### Holeboard task

#### Apparatus

**Fig. 1:**
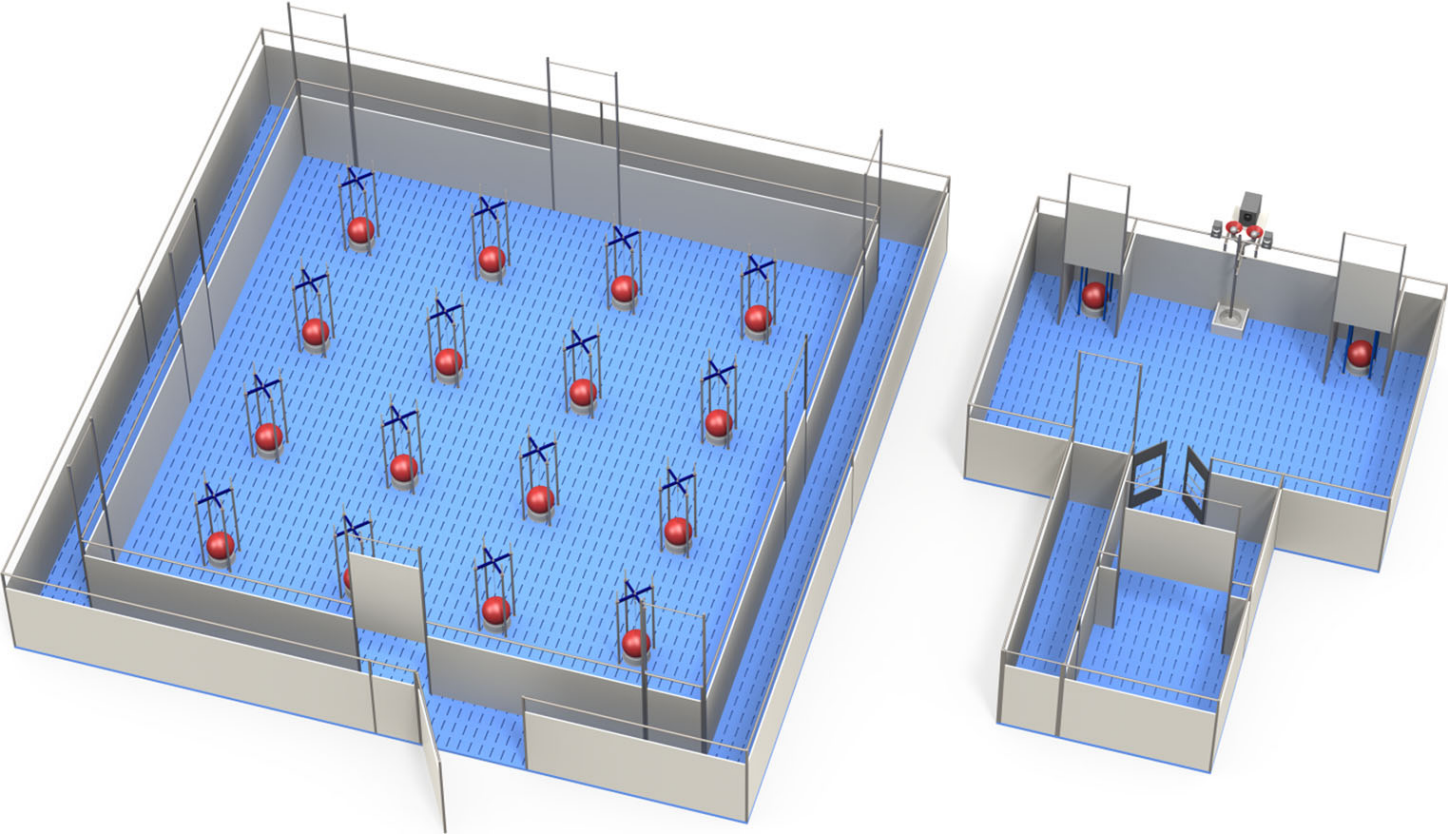
Holeboard apparatus (*left*) and judgement bias apparatus (*right*) side by side. (Illustrations by Yorrit van der Staay)

The same spatial holeboard apparatus (see Fig. 1, left panel) was used in the three studies and is described in detail by Gieling et al. (2012, 2013, 2014). The holeboard was a square arena (5.3 × 5.3 m) with 1-m-high walls. The entire arena was surrounded by a narrow corridor (40 cm wide) leading to four entrances into the arena, one in the middle of each side, which could be opened by the experimenter using pulley-operated guillotine doors. Within the arena was a 4 × 4 matrix of food bowls (Road Refresher, Jolly Pet), the ‘holes’ of the holeboard, which had a false bottom underneath which rewards could be placed to control odour cues. To control visual cues, a large hard-plastic ball (24 cm diameter) covered each food bowl. A pig could easily raise the ball off the food bowl using their snout to gain access to rewards underneath. Guide rails ensured that the ball could not be knocked off the bowl and that it returned to cover the bowl once the pig had retracted its snout. Rewards used were chocolate M&M’s^®^ (Mars Nederland b.v., Veghel, The Netherlands).

#### Habituation

Full details on habituation and training of animals for Studies 1 and 2 can be found in Gieling et al. (2013, 2014) and for Study 3 in Roelofs et al. (2017). In short, pigs were gradually exposed to the presence of experimenters and the testing apparatus during daily habituation sessions. Pigs initially explored the holeboard in groups, which gradually decreased in size until they were comfortable exploring the holeboard individually.

#### Training and testing

**Fig. 2:**
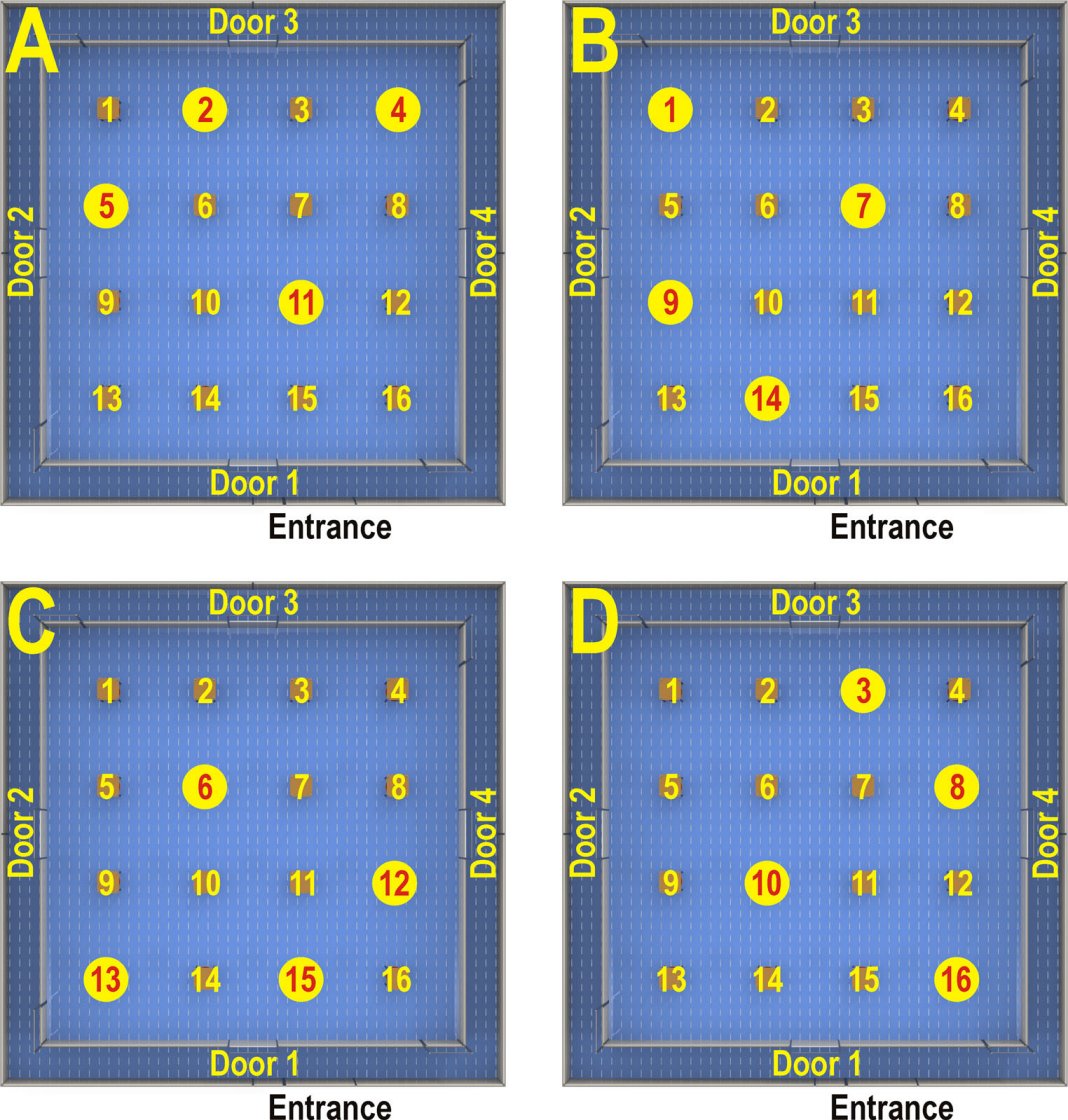
Configurations of baited holes used for the holeboard task

After habituation to the experimenter, rewards and apparatus, formal training in the holeboard began. Each animal was randomly assigned to one of four configurations of four rewarded holes (containing 1 M&M’s^®^; see Fig. 2). In each trial, a pig was let into the corridor surrounding the holeboard and walked the perimeter of the arena until it found the open entrance into the arena, the location of which was chosen randomly per trial. Pigs could then search the arena for the rewarded holes. Trials ended after all four rewards were obtained or when a maximum trial duration of 10 min (Studies 1 and 2) or 7.5 min (Study 3) had elapsed.

In each trial, working memory and reference memory were recorded. As they reduce the bias induced by incomplete trials, ratio measures for working memory and reference memory were used (van der Staay et al. 2012). Working memory was measured as the number of visits to holes which resulted in a reward (maximum of four) divided by the total number of visits to these same holes, i.e. a score of 1 would imply that a pig had not revisited any of the baited holes after obtaining a reward. Reference memory was measured as the number of visits to the baited holes, divided by the total number of visits to all holes, i.e. a score of 1 would imply that a pig had only made visits to baited holes. To get an overall measure of working memory and reference memory performance over time, data were averaged across four trial blocks. Number of trial blocks needed for acquisition of the holeboard task per study subject is provided in Table 1.

### Judgement bias task

#### Apparatus

The same judgement bias apparatus, described in detail by Murphy et al. (2013a, b, 2015), was used in all three studies (see Fig. 1, right panel). A start box (1.2 m^2^) was connected, via a small antechamber (1.2 m^2^), to a rectangular test arena (3.6 m × 2.4 m). Two goal-boxes were attached to the back wall of the test arena each of which contained a food bowl system as described above for the holeboard apparatus. Entrance to the test arena from the start box and access to each goal-box were regulated by the experimenter using pulley-operated guillotine doors. Tones were used to cue the location of rewards in the judgement bias task. Tones were generated using the open-source software Audacity (http://audacity.sourceforge.net/) and played on an MP3 player (Archos 18 Vision, 4 GB, Archos GmbH, Grevenbroich, Germany) through speakers (Logitech z-313, Logitech Europe S.A., Morges, Switzerland) attached at the back of the testing area. The training tone-cues used were a 30-s-long 200 and 1000 Hz pure tone (Waveform: Sine; Amplitude: 1). Ambiguous tone-cues were generated at equal intervals between the training tone-cues on a logarithmic scale: 299.07, 447.21, and 668.74 Hz. Rewards used were chocolate M&M’s^®^ (Mars Nederland b.v., Veghel, The Netherlands).

#### Habituation and training

Full details on habituation and training of animals for Studies 1 and 2 can be found in Murphy et al. (2013a, b) and Murphy (2015, chapter 6), and for Study 3 in Roelofs et al. (2017).

After habituation to the experimenter, rewards and apparatus, pigs were trained in a conditional discrimination task to distinguish between the two training tone-cues. In a ‘positive’ trial, a tone-cue (CS^+^) predicted a large reward (4 M&M’s^®^) in the associated ‘positive’ goal-box, while in a ‘negative’ trial a tone-cue (CS^−^) predicted a small reward (1 M&M’s^®^) in the associated ‘negative’ goal-box. Cue frequency and goal-box location in positive and negative trials were counterbalanced across animals. Each pig received one training session daily, and sessions consisted of 13 trials; three forced trials (2 negative; 1 positive), where only the correct goal-box was available, followed by 10 free trials (5 negative; 5 positive), where both goal-boxes were available but only the correct goal-box, as predicted by the tone-cue, contained a reward. Upon presentation of a tone-cue, the pig was released from the start box and had up to 30 s to choose between the two goal-boxes. A choice was defined as any lift/push of the ball covering a food bowl in a goal-box with enough force to cause the ball to move. If a pig failed to choose within this time (omission) or made an incorrect choice, both goal-boxes were closed and the pig remained in the test arena for a 90 s time-out penalty. In Studies 2 and 3, the first 3 positive and 3 negative trials in every fifth session were ‘open choice’ trials, where an incorrect choice resulted in the closing of the incorrect goal-box only and pigs could still visit the correct goal-box to collect a reward. This was used to remind pigs that rewards were available in every trial. Pigs were trained until they responded correctly four out of five times to both positive and negative tone-cues (free trials) in three consecutive training sessions. The number of sessions needed to reach this criterion of performance was taken as a measure of conditional discrimination learning.

#### Testing

Judgement bias was then assessed over four testing sessions. Each daily session consisted of 16 trials; 3 forced and 10 free trials, as before, and 3 ambiguous trials where one of the three previously unheard ambiguous tone-cues was played in lieu of one of the training tone-cues. In ambiguous trials, both goal-boxes were open. In Studies 1 and 3 neither contained a reward, while in Study 2 ambiguous trial choices were rewarded as expected, i.e. 4 M&M’s^®^ in the positive goal-box and 1 M&M’s^®^ in the negative goal-box. Once a pig had chosen a goal-box/eaten the reward, the trial was ended and the pig returned to the start box for the next trial. Each of the three ambiguous cues was presented once per day (trial numbers 6, 11 and 16). The order of trials was counterbalanced so that each ambiguous trial occurred after equal numbers of positive and negative trials. The percentage of ‘optimistic’ choices, i.e. choice for the positive goal-box, in response to each of the five cue-types (CS^−^, ambiguous cue near CS^−^, ambiguous cue intermediate between CS^+^ and CS^−^, ambiguous cue near CS^+^, and CS^+^) was calculated per pig across the four test sessions. To get an overall measure of ‘optimism’ in the judgement bias task, the unweighted mean of optimistic choice percentages in response to the individual ambiguous cue-types was calculated.

### Statistical analysis

A set of variables was selected for both the holeboard task and the judgement bias task from the three studies (see Table 2A). The variables were taken as indices of optimism, average performance level and speed of learning and subjected to correlation analysis and Bayesian analysis to evaluate whether they were independent measures; that is, whether they reflect different aspects of different cognitive processes.

**Table 2:** Means, number of animals and standard error of the mean (SEM) of the measures that were used in the correlation analyses (A). The per cent variation covered by the linear trend component calculated for all pigs of a study of acquiring the reference memory and working memory components of the holeboard task are listed in B

	Study 1			Study 2			Study 3		
	Mean	*N*	SEM	Mean	*N*	SEM	Mean	*N*	SEM
A									
HBT									
RM mean	0.480	16	0.019	0.497	37	0.013	0.535	20	0.017
RM slope	0.044	16	0.004	0.049	37	0.003	0.052	20	0.004
WM mean	0.838	16	0.015	0.837	37	0.008	0.820	20	0.012
WM slope	0.009	16	0.005	0.018	37	0.002	0.016	20	0.002
CDT									
Sessions to criterion	10.600	15	0.815	16.324	37	0.595	22.222	18	1.390
JBT									
Optimistic choice %	38.333	15	5.040	75.450	37	2.693	50.926	18	4.451

	Per cent explained variation		
	Study 1 (%)	Study 2 (%)	Study 3 (%)
B			
HBT RM lin. trend component of the within-subjects variation	98.34	99.04	96.18
HBT WM lin. trend component of the within-subjects variation	59.14	82.98	67.92

*HBT* holeboard task, *CDT* conditional discrimination task, *JBT* judgement bias task, *RM* reference memory, *WM* working memory

#### Holeboard task

**Fig. 3:**
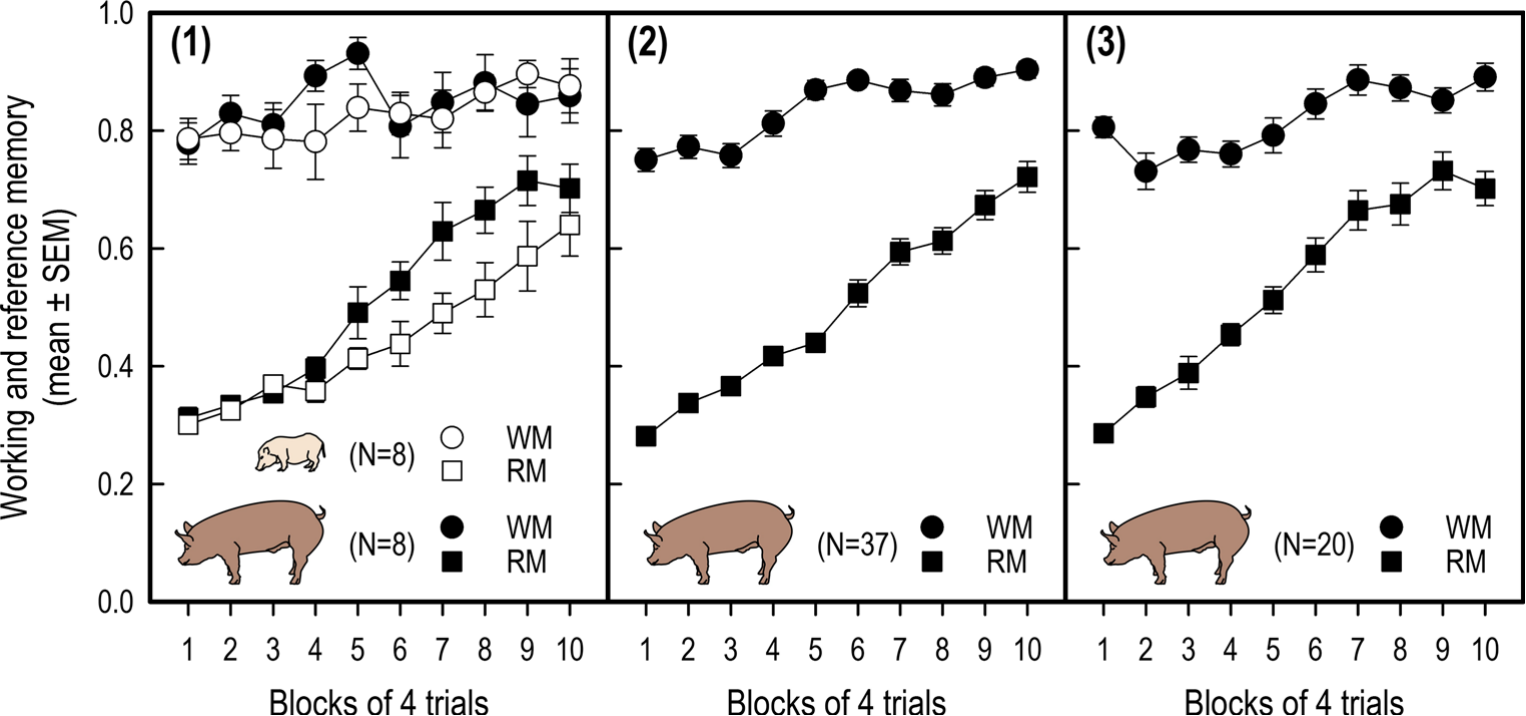
Increase in working memory (WM) and reference memory (RM) performance (mean ± SEM) across successive trial blocks in the acquisition phase of the holeboard task for studies 1–3

Although most pigs received more than 40 acquisition trials in the three studies (Table 1), we only used the first ten trial blocks (each block representing the mean of four successive trials), because pigs usually approached ceiling performance levels after 10 trial blocks, i.e. performance in later trial blocks reveals little additional information about the acquisition of the task (see Fig. 3 for working memory and reference memory learning curves per study).

For the holeboard, repeated measures analysis with the successive trial blocks as within-subjects factor was performed for each study, supplemented by polynomial contrasts (SAS PROC GLM; SAS 9.4; SAS Institute Inc., Cary, NC, USA). These orthogonal trend components define dimensions in terms of which differences in shape of learning curves can be described (Winer 1971, pp. 577–594). We determined the per cent variation explained by the linear trend component of the learning curves (Winer 1971; Cotton 1998) in the holeboard task over the successive trial blocks (van Luijtelaar et al. 1989; Spowart-Manning and van der Staay 2005) using appropriate sets of trend coefficients (from SAS PROC IML). The per cent variation in the learning curves explained by the linear trend component was calculated as the percentage of the sum of squares of the linear component of the total within-subjects sum of squares. The slopes of both working memory and reference memory calculated over the same blocks per animal, representing the linear change over blocks, were used in the subsequent correlation analysis (Table 2A). Slopes were estimated using the SAS PROC REG procedure. As representative measure of the between-subjects average performance levels, the means of the 10 successive trial blocks of working memory and reference memory were used in the correlation analysis (Table 2A).

As a considerable percentage of the variation in the increase in working memory and reference memory in the holeboard task were covered by the linear trend components (see Table 2B), the linear components (or slopes) were considered as the measures that best reflect the increase in performance across the first 10 trial blocks, i.e. the improvement can adequately be described as a linear regression line of the form: *y* = *ax* + *b* (*a*: slope, *b*: intercept). The faster the acquisition of the working memory and reference memory components of the holeboard task, the steeper the slope is. Thus, the slopes of both working memory and reference memory are representative of the overall progress of acquiring the holeboard task. The means of reference memory and working memory calculated across all trial blocks were considered as representative of overall performance level in the holeboard task. Steeper slopes will also result in a higher average performance, and consequently, slopes and mean performance may be positively correlated.

#### Judgement bias task

Two measures per pig of the judgement bias task were used for analysis: the number of trials needed to reach the learning criterion in the conditional discrimination task (sessions to criterion), and optimism (mean optimistic choice percentage across the three ambiguous tone-cues used in the judgement bias task; see Table 2A).

Shapiro–Wilk statistics (SAS PROC UNIVARIATE) confirmed that all variables of the holeboard and judgement bias task except the optimistic choice percentage of Study 2 met the criterion to be treated as being normally distributed.

#### Correlation analysis

For each study, the variables derived from the holeboard task (slopes and mean performance of working memory and reference memory) and the judgement bias task (sessions to criterion, optimistic choice percentage) were subjected to correlation analysis (SAS PROC CORR). The Pearson product-moment correlation coefficients were calculated. The optimistic choice percentage of Study 2 did not fulfil the assumption of normality. Therefore, we also determined the Spearman rank correlations (see Table 3C) between the optimistic choice percentage and the other measures in Study 2.

**Table 3:** Pearson product-moment correlation coefficients (*r*: all above diagonal) and Spearman rank correlations (*ρ*: all below diagonal), their associated *p* values, the number of animals (*N*) and Bayes factors (BF). A) Study 1; B) Study 2; C) Study 3. Correlations printed in bold italics have associated probabilities <0.05. Correlations printed in italics have associated probabilities 0.05 < *p* < 0.10. Bayes factors <0.33 (providing at least substantial evidence for HA; Wetzels and Wagenmakers 2012) or >3 (providing at least substantial evidence for* H*_0_; Wetzels and Wagenmakers 2012) are printed in bold italics

		HBT RM Mean	HBT RM Slope	HBT WM Mean	HBT WM Slope	JBT Sess. to crit.	JBT % opt. choices
**A**							
HBT RM Mean	*r*	–	***0.957***	***0.661***	0.085	0.072	0.344
	*P<*	–	***<0.001***	***0.005***	0.755	0.798	0.210
	*N*	–	16	16	16	15	15
	BF	–	***<0.001***	***0.093***	***3.096***	***3.052***	1.540
HBT RM Slope	*r*		–	***0.687***	0.260	0.037	*0.446*
	*P<*		–	***0.003***	0.331	0.895	*0.096*
	*N*		–	16	16	15	15
	BF		–	***0.065***	2.136	***3.097***	0.853
HBT WM Mean	*r*			–	0.309	−0.099	0.329
	*P<*			–	0.244	0.725	0.232
	*N*			–	16	15	15
	BF			–	1.712	2.970	1.622
HBT WM Slope	*r*				–	0.013	0.291
	*P<*				–	0.963	0.293
	*N*				–	15	15
	BF				–	***3.140***	1.896
JBT Sess. to crit.	*r*					–	0.300
	*P<*					–	0.278
	*N*					–	15
	BF					–	1.830
JBT % opt. choices	*r*						–
	*P<*						–
	*N*						–
	BF						–

B*							
HBT RM Mean	*r*	–	***0.834***	*0.308*	0.237	0.018	−0.040
	*P<*	–	***<0.001***	*0.064*	0.157	0.917	0.813
	*N*	–	37	37	37	37	37
	BF	–	***<0.001***	0.932	1.867	***4.861***	***4.756***
HBT RM Slope	*r*	***0.830***	–	0.258	0.266	0.175	0.023
	*P<*	***<0.001***	–	0.124	0.111	0.300	0.895
	*N*	37	–	37	37	37	37
	BF	–	–	1.563	1.440	2.915	***4.845***
HBT WM Mean	*r*	***0.358***	*0.295*	–	−0.233	0.024	0.042
	*P<*	***0.030***	*0.076*	–	0.164	0.889	0.805
	*N*	37	37	–	37	37	37
	BF	–	–	–	1.926	***4.841***	***4.746***
HBT WM Slope	*r*	0.174	0.231	*−0.288*	–	***0.412***	0.052
	*P<*	0.304	0.168	*0.084*	–	***0.011***	0.762
	*N*	37	37	37	–	37	37
	BF	–	–	–	–	***0.223***	***4.675***
JBT Sess. to crit.	*r*	−0.003	0.170	0.033	***0.405***	–	0.076
	*P<*	0.987	0.316	0.845	***0.013***	–	0.656
	*N*	37	37	37	37	–	37
	BF	–	–	–	–	–	***4.442***
JBT % opt. choices	*r*	−0.088	−0.073	0.108	0.026	0.023	–
	*P<*	0.604	0.667	0.524	0.878	0.892	–
	*N*	37	37	37	37	37	–
	BF	–	–	–	–	–	–

**C**							
HBT RM Mean	*r*	–	***0.750***	***0.458***	0.008	−0.187	0.005
	*P<*	–	***<0.001***	***0.042***	0.973	0.459	0.985
	*N*	–	20	20	20	18	18
	BF	–	***0.004***	0.524	***3.611***	2.658	***3.432***
HBT RM Slope	*r*		–	***0.564***	−0.079	0.120	0.189
	*P<*		–	***0.010***	0.741	0.637	0.451
	*N*		–	20	20	18	18
	BF		–	***0.158***	***3.433***	***3.094***	2.636
HBT WM Mean	*r*			–	−0.095	0.054	−0.080
	*P<*			–	0.689	0.830	0.751
	*N*			–	20	18	18
	BF			–	***3.352***	***3.360***	***3.275***
HBT WM Slope	*r*				–	0.073	*−0.426*
	*P<*				–	0.772	*0.078*
	*N*				–	18	18
	BF				–	***3.301***	0.811
JBT Sess. to crit.	*r*					–	0.354
	*P<*					–	0.149
	*N*					–	18
	BF					–	1.304
JBT % opt. choices	*r*						–
	*P<*						–
	*N*						–
	BF						–

Note that the product-moment correlations and the rank correlations are highly similar

*HBT* holeboard task, *JBT* judgement bias task, *RM* reference memory, *WM* working memory, *sess. to crit.* sessions to criterion, *% opt. choices* percentage optimistic choices

* The JBT % optimistic choices in study 2 (panel B) were not normally distributed (Shapiro Wilk test: *p* = 0.0032). Therefore, below diagonal, the Spearman rank correlations (*ρ*) are shown

The correlation analyses were performed separately for each study, because in pooled data, the differences in age and breed of the animals and modifications in the testing procedures between the three studies may artificially increase or decrease the correlations (van der Staay et al. 1990).

#### Bayesian analysis

In order to quantify the relative support provided by each study for the two competing hypotheses (i.e. whether there is (*H*_1_) or is not (*H*_0_) a correlation between the variables presented in the correlation analysis above), a Bayes factor was computed for each correlation, for each study. A Bayes factor is the ratio of the likelihood of finding the data under the conditions of the two hypotheses, *H*_1_ and *H*_0_ (Spiegelhalter et al. 2004). In this paper, we use Jeffreys’ exact solution (Jeffreys 1998; Ly et al. 2016) to compute the Bayes factors *p*(*D|H*_0_)*/p*(*D|H*_1_), based on sample size and the observed Pearson correlation coefficients. A Bayes factor larger than 1 indicates support for *H*_0_ (i.e. there is no correlation between the variables).

The computed Bayes factors were then used to calculate the probability of *H*_0_ for each possible correlation (e.g. optimistic choice and sessions to criterion, optimistic choice and mean working memory). For Bayesian hypothesis testing, a posterior odds *p*(*H*_0_ *|D*)*/p*(*H*_1_ *|D*) for the two competing hypotheses can be obtained by combining a pre-specified prior odds *p*(*H*_0_)*/p*(*H*_1_) with a Bayes factor calculated from the data. When multiple similar studies are available, under a Bayesian framework, evidence can be naturally updated by subsequently combining the studies. For each correlation, the following analysis was performed:

$${\text{Prior odds }}\times{\text{ Bayes factor study }}1 = {\text{ posterior odds study }}1$$

$${\text{Posterior odds study }}1\times{\text{ Bayes factor study }}2 = {\text{posterior odds study }}2$$

$${\text{Posterior odds study }}2\times{\text{ Bayes factor study }}3 = {\text{posterior odds study }}3$$

The prior odds represent the probability of *H*_0_ over *H*_1_ before observing the data. Before observing the data from study 1, we assigned the value 1 to the prior odds, implying both hypotheses are equally likely. The uninformative prior odds are then updated using data from the first study, resulting in posterior odds from study 1. The posterior odds from study 1 were used as informative prior odds to the Bayesian analysis of the second study. Likewise, the prior odds for the analysis of the third study are the posterior odds from the analysis of the second study. Eventually, the posterior odds *p*(*H*_0_ *|D*)*/p*(*H*_1_ *|D*) resulting from the third study can be used to compute the final posterior probabilities for *H*_0_ *: p*(*H*_0_ *|D*).

## Results

The means, number of animals and standard error of the mean (SEM) of the measures that were used in the correlation analyses are listed in Table 2A. The product-moment correlation coefficients and Bayes factors are shown in Table 3. The posterior probabilities for *H*_0_ (no correlation between variables) are shown for each correlation in Table 4.

**Table 4:** Posterior probabilities for *H*_0_ (no correlation between variables) for each correlation, based on updated Bayesian hypothesis testing using 3 separate studies. Probabilities <0.50 indicate data do not support *H*_0_, whereas probabilities >0.50 indicate data support *H*_0_

	HBT RM Mean	HBT RM Slope	HBT WM Mean	HBT WM Slope	JBT Sess. to crit.	JBT % opt.choices
HBT RM Mean	–	<0.001	0.043	0.954	0.975	0.962
HBT RM Slope		–	0.016	0.913	0.965	0.916
HBT WM Mean			–	0.917	0.980	0.962
HBT WM Slope				–	0.698	0.878
JBT Sess. to crit.					–	0.914
JBT % opt. choices						–

*HBT* holeboard task, *JBT* judgement bias task, *RM* reference memory, *WM* working memory, *sess. to crit.* sessions to criterion, *% opt. choices* per cent optimistic choices

The largest percentage of the variation in the improvement in working memory and reference memory in the holeboard task was covered by the linear trend components (see Table 2B). Therefore, the slopes of both working memory and reference memory were considered as the measures that best reflect learning across trials of the acquisition phase.

### Optimism, learning and memory

Our data strongly support the null hypothesis that optimistic choice percentage in the judgement bias task is independent of all other cognitive measures (Table 4). The posterior probabilities of *H*_0_ for individual correlations with optimistic choice percentage ranged from 0.878 to 0.962.

### Working memory, reference memory and discrimination learning

In the holeboard task, overall performance (mean) was highly correlated to task acquisition (slope) with respect to reference memory in all three studies, i.e. pigs which learned the rules of the task faster had a better overall performance in the holeboard. These findings were supported by a < 0.001 posterior probability for the null hypothesis that there is no correlation between these two variables. Overall performance and task acquisition of the working memory component were not found to be correlated in any of the studies. This was supported by a 0.917 posterior probability of independence based on the Bayesian analysis.

Our data do not fully support independence of reference memory and working memory in the holeboard task. Several correlations were found between the slope of the working memory learning curve and both reference memory measures (Table 3). Bayesian analysis provided posterior probabilities of 0.043 and 0.016 for independence between these measures, suggesting our data provide strong evidence for correlations between working memory acquisition and both measures of reference memory in the holeboard task. However, for independence of mean working memory of both reference memory measures, posterior probabilities were >0.9, suggesting there is no correlation (Table 4).

Our data support the null hypothesis of no correlation between learning in the conditional discrimination task (sessions to criterion) and performance in the holeboard (working/reference memory slopes and mean performance). Posterior probabilities for independence ranged from 0.698 to 0.980 (Table 4).

## Discussion

The present paper explored the (in)dependence of pigs’ performance in a judgement bias task (optimism measured as mean optimistic choice percentage), a holeboard task (spatial learning measured as reference memory and working memory) and a conditional discrimination task (learning measured as sessions to criterion) preceding testing in the judgement bias task. As expected, data from three independent studies provided evidence that optimism in the judgement bias task is independent of cognitive abilities assessed in the holeboard task and conditional discrimination task. Contrary to expectation, our data do not support the hypothesis that working memory and reference memory are entirely independent measures. Finally, although a correlation between reference memory in the spatial holeboard task and performance in the auditory conditional discrimination task was expected, our data support the notion that these measures are independent.

### Optimism, learning and memory

One of our criteria for behavioural tests of emotions in pigs is that ‘(…) the task should specifically and unambiguously capture emotion-related behaviours. For example, results can be confounded by differences in learning capacity (…)’ (Murphy et al. 2014, p. 12). One approach to assessing the construct validity of our judgement bias task is therefore to test the independence of responses to ambiguity from potential confounding factors such as learning ability. In the present study, we aimed to investigate whether ‘optimism’ in a judgement bias task, reflected by expectation of positive outcomes, was affected by differences in learning ability as measured by reference and working memory performance in a spatial holeboard task and by the sessions to criterion in the auditory conditional discrimination training prior to the judgement bias task in three pig studies. Validating the results of judgement bias tasks is of importance, as they are increasingly used to assess emotional state in animals (Roelofs et al. 2016).

Many studies have attempted to assess the predictive validity of the judgement bias paradigm through the effects of treatments assumed to influence mood. For example, enrichment produces a more optimistic judgement bias in both pigs and rats (Brydges et al. 2011; Douglas et al. 2012), while chronic stress (induced pharmacologically or by repeated restraint) produces a more pessimistic judgement bias in rats (Enkel et al. 2010; Rygula et al. 2013). However, few studies have attempted to assess what other processes may impact upon responses to ambiguity. We have previously demonstrated that optimism in the same judgement bias task as used for the current study was not related to decision-making under risk in a gambling task in pigs (Murphy et al. 2015). Similarly, Bateson et al. (2015) stated that responses to ambiguous stimuli in a judgement bias task in another species, the European starling, did not reflect their cognitive abilities. They based this conclusion on their finding that while experimental treatment affected judgement bias, it did not affect operant or discrimination training prior to judgement bias testing. Such dissociation in effects on task acquisition and judgement bias has been reported more often (e.g. Enkel et al. 2010; Parker et al. 2014; Gordon and Rogers 2015; Brajon et al. 2015). Several studies specifically report a lack of correlation between performance during discrimination training and measured judgement bias as indication that they are independent (Murphy et al. 2013b; d’Ettorre et al. 2016). Such correlation analysis has also been extended to other cognitive tasks. For example, no correlation was found between judgement bias and performance in simple maze tasks in sheep (Destrez et al. 2013; Coulon et al. 2015).

Although these findings, taken together, can be taken as support for the hypothesis that results of judgement bias tasks are not confounded by cognitive abilities, none of these studies provide a measure of support for the null hypothesis. To do this, Bayesian analysis is required (Wagenmakers et al. 2016). Therefore, the current study goes one step further towards determining whether judgement bias is a measure independent of learning and memory. It is the first to quantify evidence for the null hypothesis that optimism in the judgement bias task, learning and memory in the spatial holeboard task and acquisition of an auditory conditional discrimination task reflect different cognitive domains in the same animal. Based on this study, our active choice judgement bias task is not confounded by pigs’ cognitive abilities.

### Working memory, reference memory and discrimination learning

In the current study, we could not provide support for the hypothesis that working memory and reference memory are independent. Although the acquisition of the working memory component was independent of both measures of reference memory, the overall working memory performance was related to reference memory slope and overall performance. This finding is in contrast with previous studies, which have shown that working and reference memory in the holeboard can be influenced independently of each other by experimental manipulations. For example, when assessing the effects of environmental enrichment on working and reference memory of pigs in a holeboard, Bolhuis et al (2013) found that reference memory was unaffected by enrichment, whereas it improved working memory. Similar results have been found for rodents, where either reference or working memory was affected by experimental treatment (Blokland et al. 1998; Prickaerts et al. 1999; Kuc et al. 2005; Bainbridge et al. 2008). Also, reference memory and working memory are affected differently by chronic stress (Conrad 2010). It is possible that we found working and reference memory to be correlated due to our testing conditions. There was no lasting effect of treatment on working or reference memory in any of the studies used (Gieling et al. 2013, 2014; Roelofs et al. 2017). It is likely that when unaffected by treatment, pigs show unimpeded improvement in both working and reference memory, resulting in a positive correlation between these measures.

While both measures used to represent reference memory were highly correlated, our data suggest that the acquisition of the working memory component and overall working memory performance were independent. This unexpected finding was likely due to a lack of improvement shown in the learning curves of working memory. Some pigs already demonstrated a high level of working memory performance from the start of the holeboard task. The working memory component of the holeboard task is based on natural foraging behaviour using a win-shift foraging strategy (Gustafsson et al. 1999); pigs have previously been shown to acquire a win-shift task faster and perform it more accurately than a win-stay task (Laughlin and Mendl 2000). This could explain why working memory learning curves for pigs display a ceiling effect quite quickly. Working memory slope may therefore not be as useful a measure to describe working memory learning in pigs as it is for other species with a steeper learning curve (e.g. mice; Kuc et al. 2005).

### Discrimination task and reference memory

Interestingly, auditory conditional discrimination learning in the judgement bias task, a task which entails an element of spatial discrimination and where rule learning is important for successful performance, was unrelated to reference memory in the holeboard task, a task which entails a more complex spatial discrimination. Correct responding in the conditional discrimination task, however, may not entirely reflect reference memory capacity. Pigs which took longer to learn may have struggled to accept the inequality of reward between positive and negative trials, rather than failing to remember the rules of the task. Capuchins show more refusals when offered a less preferred food in the presence of an unobtainable preferred food (Dubreuil et al. 2006). It is possible, therefore, that the pigs took some time to understand that when the small reward is signalled, the large reward is not available and that longer learning times also reflect greater frustration at the perceived inequality rather than purely discrimination abilities. This is supported by the fact that before pigs reach criterion on the conditional discrimination task in the judgement bias task, their latencies to respond in negative trials increase (own, non-systematic observations), suggesting they are already aware of the association between cue and reward size. Similarly, monkeys showed shorter response times in an operant task when preferred rewards were signalled than when less preferred rewards were signalled (Watanabe et al. 2001). The conditional discrimination task used prior to judgement bias testing, as designed, does not allow us to distinguish between discrimination learning where correct choices are equally rewarded and discrimination learning when there is inequality between the choices.

### Conclusions

The present study provides support for the notion that optimism measured in the judgement bias task is unrelated to the animals’ cognitive abilities in the holeboard task and in the conditional discrimination task. Based on three separate studies, evidence was provided that optimism in the judgement bias task on the one hand, and working and reference memory performance in the holeboard task and the acquisition of the conditional discrimination task preceding judgement bias testing on the other, were independent. These results further validate the use of judgement bias as a proxy measure of emotional valence in animals.
